# Cervical ectopic pregnancy after *in vitro* fertilization: case report successfully treated with cervical electric aspiration

**DOI:** 10.5935/1518-0557.20190036

**Published:** 2019

**Authors:** Jefferson Drezett, Dinah Marques, Roberta Ottoboni, Artur Dzik, Mario Cavagna

**Affiliations:** 1 Reproduction Department of Pérola Byington Hospital

**Keywords:** Ectopic pregnancy, Cervix uteri, *In vitro* fertilization, Multifetal pregnancy reduction

## Abstract

Cervical ectopic gestation is a serious and potentially lethal condition considered exceptional in in vitro fertilization. Early diagnosis is critical to successful treatment and preservation of fertility. We report a rare case of cervical pregnancy after in vitro fertilization and embryo transfer, successfully treated exclusively with electrical aspiration. Case report: A 36-year-old patient attended the Department of Human Reproduction at Pérola Byington Hospital in 2015 due to primary infertility with no apparent cause for seven years. Subjected to ovulation induction with recombinant depot FSH and GnRh analogue, triggered with chorionic gonadotropin. It evolved with the collection of ten oocytes and transfer of two embryos, with cryopreservation of the remaining ones. The control with serial ultrasound showed a gestational sac in uterine cervix topography, indicating gestation of six weeks, confirmed after 24 hours by a second operator. The treatment was successfully performed by electric aspiration with an EasyGrip^®^ cannula of 6 mm in diameter, without the occurrence of hemorrhage or the need for other procedures. Early diagnosis allowed successful conservative treatment with only cervical aspiration. The literature review confirms the rarity of the case, but does not indicate consensus on the best treatment of cervical ectopic pregnancy.

## INTRODUCTION

Cervical pregnancy is a rare and potentially lethal form of ectopic gestation, with incidence between 1:2,500 and 1:12,000 clinical pregnancies (Anev *et al*., 2013; Jozwiak *et al*., 2003; Lin *et al*., 2013). The occurrence of cervical pregnancy has increased in recent decades with the increasing use of assisted reproductive techniques, although the causes are not sufficiently clarified (Anev *et al*., 2013; Chen *et al*., 2001; Lesny *et al*., 1999; Weyerman *et al*., 1989).

In the clinical diagnosis of cervical pregnancy, the uterus may be hourglass-shaped, indicating disproportion between the size of the body and the cervix. In a few cases it is possible to visualize the gestational content in the specular examination when there is dilatation of the cervix. These findings, however, correspond to the late diagnosis, generally after the 10^th^ week of gestation, when there is a greater risk of hemorrhage and hypovolemic shock, or the need for emergency hysterectomy (Chen *et al*., 2001).

Ultrasonography is essential to confirm early cervical pregnancy, significantly increasing the possibility of preserving the reproductive capacity of the woman. The treatment of cervical ectopic pregnancy is admittedly difficult and there is no consensus on the best approach (Lesny *et al*., 1999). The success of the treatment is reported with different interventions such as cervical suture, cerclage with uterine curettage, cervical balloon tamponade, uterine artery embolization, methotrexate, potassium chloride injection or hysteroscopic resection (Jozwiak *et al*., 2003; Chen *et al*., 2001; Prorocic & Vasiljevic, 2007; Bennett *et al.*, 1993).

Despite these alternatives, the diagnosis is rarely early enough so that the treatment can be done through aspiration of the pregnancy without the occurrence of intense bleeding or the need for emergency hysterectomy. Thus, the objective of this article is to describe a rare clinical case of cervical ectopic pregnancy after in vitro fertilization (IVF) with embryo transfer (ET), successfully treated with exclusive electric aspiration of the cervical canal. Because it was a case report, it was not necessary to submit to the Research Ethics Committee or to adopt a Free and Informed Consent Form.

## CASE REPORT

A 36-year-old patient admitted to the Human Reproduction Department of Pearl Byington Hospital in March 2015 for primary infertility seven years ago. Menarche at eleven years, with regular cycles of four days and interval of 28 days. For seven years, she had had three intercourse sessions per week without the use of a contraceptive method. A history of hyperprolactinemia with nuclear magnetic resonance without torsal seizures in 2012, treated with cabergoline for two years with normalization of prolactin levels.

Physical examination at admission showed good general and nutritional status. Transvaginal ultrasonography indicated 43 cc uterus, 7 mm intramural myoma and normal volume and appearance ovaries. Oncotic colpocitology without neoplastic alterations or inflammatory condition. Hysterosalpingography showed patent tubes with no abnormalities. Hemogram with Hb 10.2 g/dl, leukocytes 5,160 thousand/mm^3^, 542 thousand platelets; creatinine 0.7 mg/dl; glucose 99 mg/dl; TSH 1.65 mIU/L; Free T4 0.82 ng/dL; prolactin 15.6 ng/dL; estradiol 34 pg/ml; CA 125 14.8 U/mL. Serologies for hepatitis B, hepatitis C, syphilis, HIV and rubella were negative.

The patient underwent ovulation induction, initiated on the third day of the cycle with recombinant depot FSH (Elonva^®^ 150 mcg) and GnRh analog (Orgalutran^®^ 0.25 mg) for six days, with follow-up of follicular development by serial transvaginal ultrasonography. On the second day of the cycle, the right ovary had four antral follicles, the left ovary with six antral follicles and an endometrial echo of 5.9 mm. At the end of the induction showed three follicles above 18 mm in the right ovary and another five follicles of similar measurements in the left ovary. Triggered with chorionic gonadotrophin (Choriomon^®^ 5,000 IU).

Subjected to follicular aspiration with total collection of ten oocytes. A conventional IVF was performed, with a total of nine embryos, and two embryos were transferred to D3 with a Sydney catheter, without the use of tweezers for neck extension, scarce blood in the catheter and absence of embryo retained after transfer. Seven embryos were cryopreserved.

**Figure 1:**
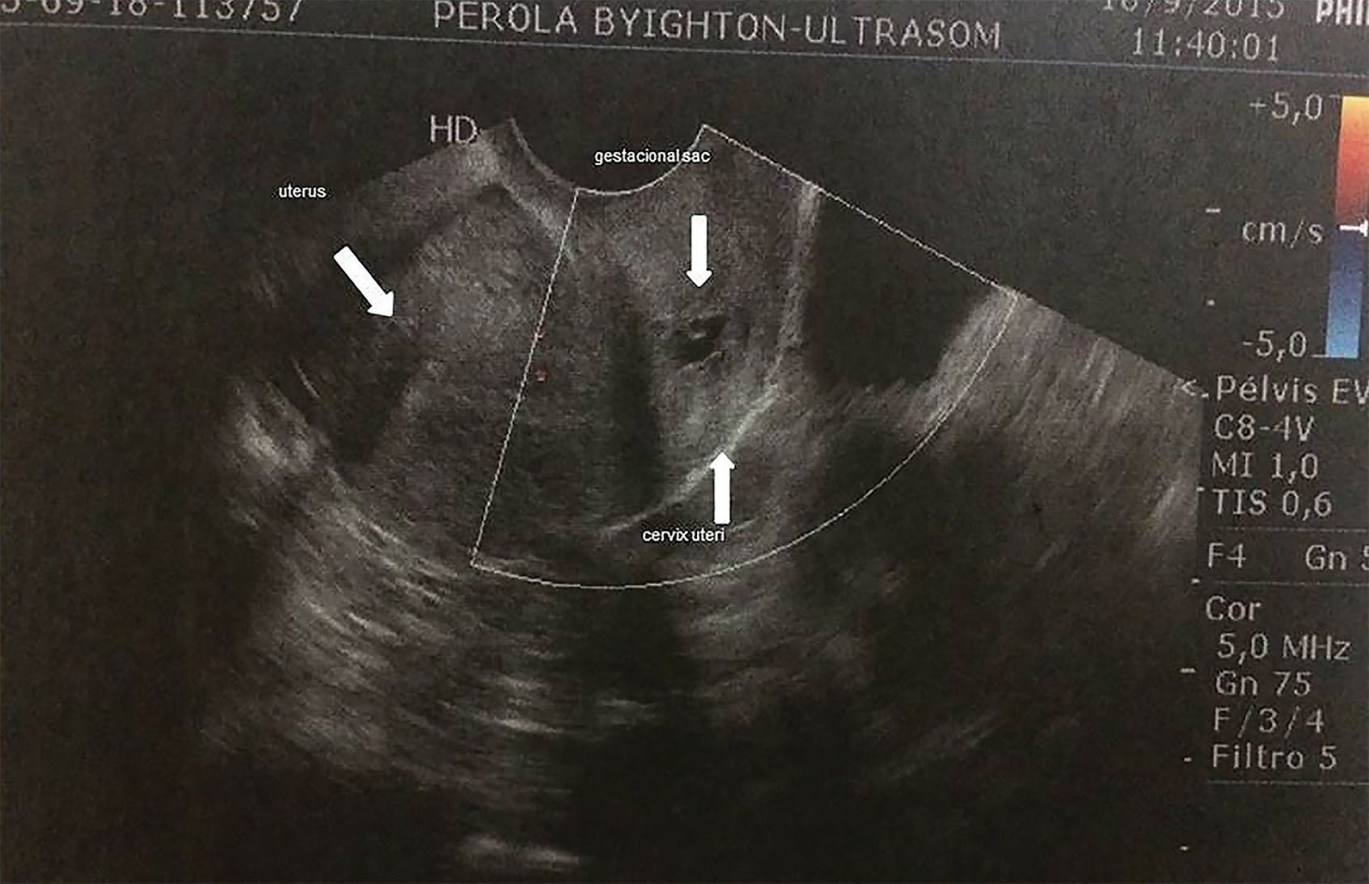
Transvaginal ultrasonography identifying the ectopic pregnancy presenting gestational sac in cervical topography and empty uterine cavity

**Figure 2:**
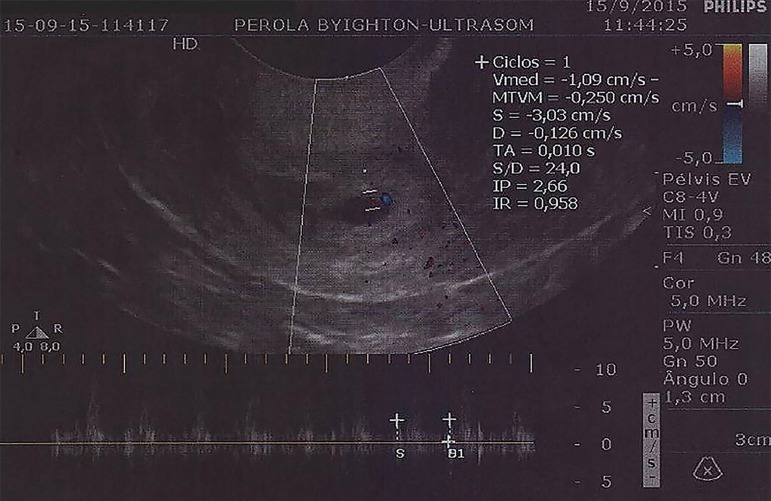
Detail on transvaginal ultrasonography of cervical ectopic gestation

Patient presented dosage of beta hCG 4,815.93 IU/mL on the 26^th^ day after ET. At 33 days ET had vaginal bleeding. Transvaginal ultrasonography showed cystic image measuring 4.9 mm in the cervix, compatible with gestational sac with 3 mm embryo, indicating cervical gestation of 6 weeks (Figures 1 and 2). It was observed peripheral and parietal neovascularization with high resistivity index. The test was repeated after 24 hours by another operator, confirming the diagnosis.

Laparoscopic emptying of the cervix with a 6 mm diameter Karman cannula (EasyGrip^®^ cannula 6 mm) was performed without the use of ultrasonography. No cervical canal tamponade was required due to scarce cervical bleeding at the end of the operative period. Patient was kept hospitalized for 24 hours for strict observation of vaginal bleeding. She was discharged in good clinical condition, without vaginal bleeding and without the need for other interventions. She returned after a week to review the procedure, without vaginal bleeding and with normal ultrasound examination.

## DISCUSSION

The review of the literature in the database of the US National Library of Medicine National Institute of Health (PubMed) using the descriptors ("Fertilization in Vitro" [Mesh]) and "Pregnancy, Ectopic" and "Cervix Uteri" Mesh] results in 25 articles published in the last 32 years, indicating the rarity of cervical ectopic pregnancy after IVF.

The literature indicates a frequent association of cervical ectopic pregnancy after IVF-ET with heterotopic pregnancy. This form of ectopic pregnancy is defined by concomitance with another intrauterine pregnancy, a rare event in spontaneous pregnancies estimated at 1: 30,000 pregnancies. However, in the last three decades this rate has been increasing significantly with assisted reproduction techniques (Jozwiak *et al*., 2003; Lin *et al*., 2013).

The etiology of cervical gestation after IVF-ET is poorly understood. It is believed to be related to the exacerbation of contractions of the junctional zone of the uterus in the luteal phase as a consequence of the elevation of progesterone, producing a similar effect to that occurring on the tubal motility. It is possible that the uterine fundus stimulation provoked by the probe during ET also changes the contractility of the junctional zone. In addition, women who attended other forms of ectopic pregnancy had a higher peak of estradiol after ET (Lesny *et al*., 1999).

Authors such as Bennett *et al.* (1993) and Yu *et al.* (2014) accept these hypotheses, although they warn that women who undergo assisted reproduction procedures and who undergo cervical ectopic pregnancy often have other recognized risk factors, such as previous uterine instrumentation, uterine and cervical anatomical abnormalities, Asheman's syndrome, uterine fibrosis, chronic endometritis or endometrial atrophy.

ET timing does not appear to be associated with an increased risk of cervical ectopic pregnancy (Lesny *et al.*, 1999). However, authors suggest that the difficulty of ET or manipulation of the uterine cervix increases the risk of ectopic pregnancy, either through the use of dilators, rigid probes for wire transfer or guidewire, or neck elongation clamps (Bennett *et al.* 1993; Lesny *et al.,* 1999; Chen *et al.,* 2001). Considering these possibilities, Yovich *et al.* (1985) recommend that the ET catheter be inserted 55 mm routinely. In this case report, the patient had no known risk factors for cervical ectopic pregnancy, no dilation or elongation of the cervix was used, and the ET presented no difficulty.

The literature reports 12 cases of exclusive cervical ectopic pregnancy after IVF-ET (Weyerman *et al*., 1989; Bennett *et al*., 1993; Ginsburg *et al.,* 1994; Saliken *et al.,* 1994; Pattinson *et al.,* 1994. Two cases of twin cervical ectopic pregnancy, rarer, are reported by Anev *et al.* (2013) and by Aboulfoutouh *et al.* (2011). In all other cases, cervical ectopic pregnancy after IVF-ET was heterotopic. Other exceptional situations are described by Lin *et al.* (2013), in case of triple heterotopic pregnancy after IVF-ET, with topical, tubal and cervical embryo, and by Davies *et al.* (1990), who describe cervical heterotopic gestation in a patient with previous cervical ectopic pregnancy.

According to Chen *et al.* (2001), hysterectomy remained until the early 1980s as the main treatment of cervical ectopic pregnancy, definitively compromising the woman's reproductive future. Ultrasonography changed this scenario, reducing maternal mortality in cases of cervical ectopic pregnancy from 40% to less than 10%. When performed before the 10^th^ week of pregnancy, ultrasonography makes the early diagnosis of cervical ectopic pregnancy. This allows more effective interventions that significantly reduce the risk of bleeding and death, as well as maintaining the reproductive capacity of women (Bennett *et al.,* 1993). Magnetic resonance imaging has rarely been used in the propaedeutics of cervical ectopic pregnancy and should be considered, as advocated by Pattinson *et al.* (1994) and Ginsburg *et al.* (1994).

In the last decades conservative treatments have been described with success, mainly in the heterotopic gestations in which it is intended to maintain intrauterine gestation. Only one case of spontaneous abortion of cervical heterotopic pregnancy with normal follow-up of intrauterine pregnancy is reported by Livingstone *et al*. (2000). Anev *et al.* (2013) believe that the method should be decided together with the woman, considering her desire for future pregnancy and the experience of the medical staff. Retrospective study by Yu *et al.* (2014) with 25 heterotopic pregnancies of different types showed that most cases of heterotopic cervical pregnancy were treated with local injection of methotrexate, combined or not with the injection of potassium chloride.

Cho *et al.* (2007) reported a case of aspiration of cervical ectopic pregnancy after 7 weeks IVF-ET guided by ultrasonography, maintaining the gestation until the 35^th^ week. Similar treatment was adopted by Hsieh *et al.* (2008), with transvaginal aspiration of the cervical embryo guided by ultrasonography preserving intrauterine gestation. Chen *et al.* (2001) also chose the aspiration of cervical pregnancy after IVF-ET, but associated the suture of the neck to prevent hemorrhage, preserving the topical pregnancy until the term.

Some authors adopt more complex treatments in more adverse circumstances. Tsakos *et al.* (2015) report cases of heterotopic cervical pregnancy with live embryos after oocyte donation IVF-ET, successfully treated with cervical aspiration followed by placement of Foley catheter and cerclage. Topical pregnancy followed without complications with elective cesarean delivery at week 38. In another case, Prorocic & Vasiljevic (2007) were able to preserve an intrauterine twin pregnancy by treating cervical pregnancy with aspiration and injection of hypertonic solution of KCL after ligation of descending cervical branches of the uterine arteries.

The exclusive use of the KCL injection for embryo reduction in a case of cervical ectopic pregnancy was an alternative of Carreno *et al.* (2000), resulting in topical gestation up to the 36^th^ week with a healthy newborn. Honey *et al.* (1999) used the same strategy for cervical embryo reduction, preceded by selective fluoroscopic embolization of the uterine arteries for prophylaxis of cervical hemorrhage. Although they have been successful in the treatment of cervical pregnancy, adverse events have not allowed the evolution of topical pregnancy until the term.

Hysteroscopic resection was alternative to Jozwiak *et al.* (2003) to treat cervical heterotopic pregnancy and maintain intrauterine pregnancy to term successfully. The use of methotrexate for the treatment of cervical heterotopic gestation is described by authors such as Bratta *et al.* (1996), Hsieh *et al.* (2004), Aboulfoutouh *et al.* (2011), Sánchez-Ferrer *et al.* (2011) and Tsakos *et al.* (2015). The association of methotrexate with embolization of the uterine arteries and cervical aspiration was adopted by Sánchez-Ferrer *et al.* (2011).

The few cases of exclusive cervical ectopic pregnancy after IVF-ET described in the literature were treated with different therapeutic approaches. Uterine artery embolization was employed by Saliken *et al.* (1994) as a preoperative measure. Subsequently, Yu *et al.* (2009) opted for the same procedure before cervical emptying, also achieving success. Two cases of cervical gestation after IVF-ET were reported by Bennett *et al.* (1993), both successful with aspiration of pregnancy followed by tamponade of the cervix with balloon.

Other forms of treatment of cervical ectopic pregnancy are described. Hsieh *et al.* (2004) opted for the use of methotrexate and intracervical injection of vasopressin. In an unusual case, Anev *et al.* (2013) describe a diamniotic and monochorionic twin cervical ectopic pregnancy after single ET, diagnosed at week 6 and treated with the combination of mifepristone and systemic methotrexate, followed by aspiration cervical emptying. Aboulfoutouh *et al.* (2011) also reported a case of twin cervical ectopic pregnancy after IVF-ET, but opted for ultrasound-guided aspiration and single methotrexate systemic injection. Sieck *et al.* (1997) have been successful in employing exclusive methotrexate to treat cervical ectopic pregnancy. Similar procedure was adopted by Piccioni *et al.* (2015), which made possible later pregnancy to term.

Few cases of second trimester cervical pregnancy are described. The first case of second trimester cervical pregnancy after IVF-ET was described by Weyerman *et al.* (1989), completed at the 26^th^ week of gestation after hysterectomy, with a newborn of 830 grams. Pattinson *et al.* (1994) report the treatment of this condition with embolization of the uterine arteries followed by emptying of the cervix and placement of an intracervical balloon. Successful conduct allowed for new ET to be performed, resulting in uncomplicated pregnancy and delivery with healthy newborn.

## CONCLUSION

Cervical ectopic pregnancy after IVF-ET is a rare condition. The different approaches found in the literature indicate that there is no consensus on the best treatment. This report is the only case of successful treatment of cervical pregnancy after IVF-EF only with the use of cervical aspiration. The experience of different authors is unanimous in considering the fundamental routine ultrasonography after IVF-ET for the early diagnosis of exclusive or heterotopic cervical ectopic gestation, significantly reducing the risk of adverse events.
